# Supplementary material for: Vitamin B12 Levels, Substance Use Patterns and Clinical Characteristics among People with Severe Substance Use Disorders: A Cohort Study from Western Norway
Source: Nutrients. 2022 May 5;14(9):1941. doi: 10.3390/nu14091941 (PMC9105230; doi:10.3390/nu14091941)
Supplement: Supplementary file 1 [file nutrients-14-01941-s001.zip › nutrients-1676414-supplementary.pdf]

Table S1. Mean corpuscular volume among participants with sufficient, insufficient, and deficient levels of vitamin B12.

Vitamin B12 status	Mean corpuscular volume Median (IQR)
Adequate (>300)	91 (87–95)
Suboptimal (175–300)	91 (88–94)
Deficient (<175)	95 (89–99)

The table displays median (IQR) mean corpuscular volume among participants with sufficient (n = 504), insufficient (n = 133) and deficient (n = 8) vitamin B12 levels at baseline.
